# HTrack: A new tool to facilitate public health field visits and electronic data capture

**DOI:** 10.1371/journal.pone.0244028

**Published:** 2020-12-15

**Authors:** Dania M. Rodríguez, Kyle Ryff, Liliana Sánchez-Gonzalez, Vanessa Rivera-Amill, Gabriela Paz-Bailey, Laura Adams

**Affiliations:** 1 Division of Vector Borne Diseases, Centers for Disease Control and Prevention, San Juan, Puerto Rico; 2 Ponce Health Science University-Ponce Research Institute, Ponce, Puerto Rico; Chiang Mai University Faculty of Medicine, THAILAND

## Abstract

Many applications have been developed for electronic data collection. However, offline field navigation tools incorporating secure electronic data capture and field visit tracking are currently scarce. We created an R-Shiny application, HTrack (Household Tracking), for use on encrypted Android devices in the field. The application was implemented in the Communities Organized to Prevent Arboviruses (COPA) project, a study beginning in 2018 to better understand arboviral disease incidence in 38 communities in Puerto Rico. The application was used to navigate to randomly selected structures and capture visit outcomes after conducting multiple visits for participant recruitment. It also served as a bridge to an alternate software, Epi Info, to collect participant-level questionnaire data. This application successfully captured each visit outcome and improved the logistics of field level activities for the COPA project, eliminating the use of paper maps for navigation. We show the development of HTrack and comment on the limitations and strengths of this application and further improvements.

## Introduction

Community-based studies often require navigating to and around field sites dynamically to identify selected households, monitor contact attempts, and capture visit outcomes. These activities require specific software tools that allow for electronic data capture at the field-level. The development of these tools has increased in par with different research needs, including the incorporation of new features and advancements. However, selection or creation of a specific software tool requires a careful assessment of the study needs and an evaluation of the corresponding tools offered by different programs.

Many software tools are available for different aspects of field data collection and navigation. For example, the Environmental Systems Research Institute (Esri) provides a powerful suite of applications developed for mapping, spatial analysis and field operations [1–4]. This is a robust suite that can collect field-level data online or offline and perform field navigation as needed [5]. Its use must be paired with ArcGIS Online and ArcGIS Server [6, 7], where the user needs an Esri institutional account to be able to use the application. However, stringent data security requirements from some agencies, including the U.S. government, restrict the storage of identifiable information on external servers including Esri’s cloud computing environment. Another example is Epi Info, an application developed by the Centers for Disease Control and Prevention (CDC). This tool was built to enable epidemiologists and other health professionals to collect and analyze survey data in the field in both online and offline settings, though it lacks field navigation capacity [8–10]. Several other software tools have been reviewed in an effort to consolidate information related with their availability and provided features associated with offline field-level data capture [5, 11–13]. However, the features provided by these tools either do not always include built-in navigation capabilities nor comply with stringent data security requirements, particularly for government users. This often results in the use of paper maps, multiple devices or tools, or staff knowledge of the area, all of which can be challenging or logistically impossible for large studies.

### Communities organized to prevent arboviruses (COPA)

COPA is a community-based cohort study initiated in 2018 to assess arboviral disease incidence in Ponce, Puerto Rico. To enable electronic data capture, multiple software programs were evaluated to identify the best option for field use; however, none of the tools assessed met all project needs. This study involved the selection and recruitment of a random sample of >3800 participants in 38 communities consisting of several neighborhoods and public housing areas with apartment building complexes. Field staff were required to visit all selected households up to three times on different days and record all visit statuses and outcomes, including re-scheduling visits for future dates. Limited cellular connectivity in the study areas, as well as strict data security requirements, resulted in the need for an offline tool that would perform site navigation and field-level data capture. To meet these requirements, we created an offline, R-Shiny Household-Tracking application (app), HTrack, for use on Android devices in the field. The app includes a live map that provides GPS (Global Positioning System) navigation using pre-loaded maps, a data entry area, and a searchable data table that can be pre-populated and customized to provide field-level information. Data were stored locally on the device and the map updated in real-time, enabling teams to see their current progress and pending visits for the day. Participant interviews were captured using Epi Info, and HTrack incorporated a feature that linked specific structure IDs to the corresponding Epi Info case.

We describe the development, dependencies, and limitations of HTrack in both neighborhood and apartment complex areas and provide field-level results from its use for the COPA project.

## Methods

### HTrack development

HTrack was written in R 3.3.2 (R Foundation for Statistical Computing [14]) as a Shiny application [15, 16]. This app was developed in Windows 10 for use on Android tablets and incorporates other programming languages including JavaScript, Python, and HTML to facilitate integration with the Android system. Currently, the app is not supported in iOS systems.

### Dependencies and availability

To use HTrack on Android devices, an Operating System (OS) to run R was needed. Currently, R can be installed in Windows OS, Mac OS or Linux OS. However, these are not available on most mobile devices. For Android devices, R was installed via an app called UserLand (v. 2.7.2) [17], an open-source app developed to install several Linux distributions to the device without rooting the device, which was important to maintain the security features required for the project. To streamline and simplify the app launching process, we created several shell scripts that would allow for HTrack to be started through a one-word command. Once started, the app was hosted on an offline local server. An app called DroidScript [18] was used to open that server in the device’s default internet browser (Google Chrome is recommended). Another supporting app, GPS Connected [19], was used to lock the GPS signal of the device while HTrack was used in the field; avoiding loss of GPS signal while moving around different field sites or working in areas where satellite signal was low.

This application was developed for offline use in encrypted Android tablets as needed for the COPA project. However, it can be easily modified to work online on both Windows or Android based devices for any field studies requiring house tracking or site-based data collection. All source code is available at GitHub, https://github.com/dmrodz/htrack. This repository also contains all dependencies and resources needed to be able to setup, run, customize and troubleshoot HTrack. Literacy in R and programming languages mentioned above, together with literacy in different Operating Systems is needed for customization of this application.

### Mapping component and data pre-load

A list of coordinates with unique structure IDs and community IDs was used as a pre-load file (.csv) for HTrack. The pre-load file was generated using ArcGIS Desktop [20], where community boundaries were drawn around areas that contained residential structures eligible for recruitment. Individual structures were geo-coded using aerial imagery and visual observation in the field. Structures within each community area were assigned a unique ID (HHID, Household ID), and latitude and longitude of the centroid of each structure were calculated to guide field navigation. In each community, we randomly selected a sufficient number of geocoded structures in order to reach a target of 100 participants per community.

Using coordinates surrounding the communities of interest, map tiles from Open Street Maps (OSM; x_y_z format) were generated in R using the RgoogleMaps package [21]. These were stored in each tablet in local servers coded in Python [22] and were used by the R leaflet package [23, 24] to generate an offline, interactive map for HTrack. The pre-load file was then loaded into the tablets to populate the map with house markers associated with the corresponding coordinates.

Household recruitment estimates were continuously monitored to determine if the target number of participants recruited was reached. If field staff were unable to recruit the needed participants after all structures had been visited, a new selection of randomly selected structures was generated. These were imported to HTrack through a built-in, limited access feature that merged the most recent data stored in the tablet with the new selection of structures. This allowed project planners to guide field staff to the new structures while avoiding previously visited households.

### Main user interface

**Fig 1:**
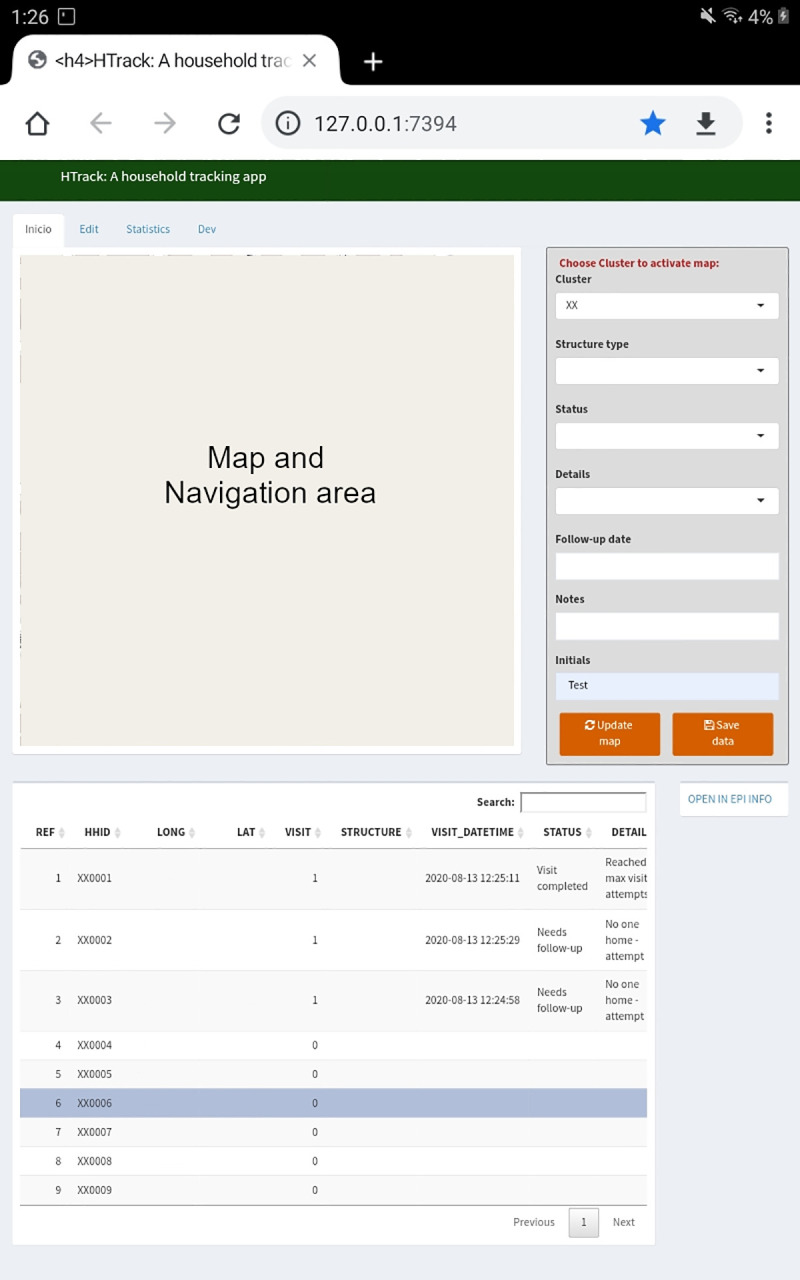
HTrack main tab serves as the general area for site navigation and data entry. This tab incorporates a live map (not shown) with zoom capabilities and a location/navigation toggle (current location appears as a red dot, not shown). The box to the right is the data entry component and is activated by pressing a house marker on the map. The data table below is populated upon data entry and saving. Coordinates are removed from the image to protect participant privacy.

**Fig 2:**
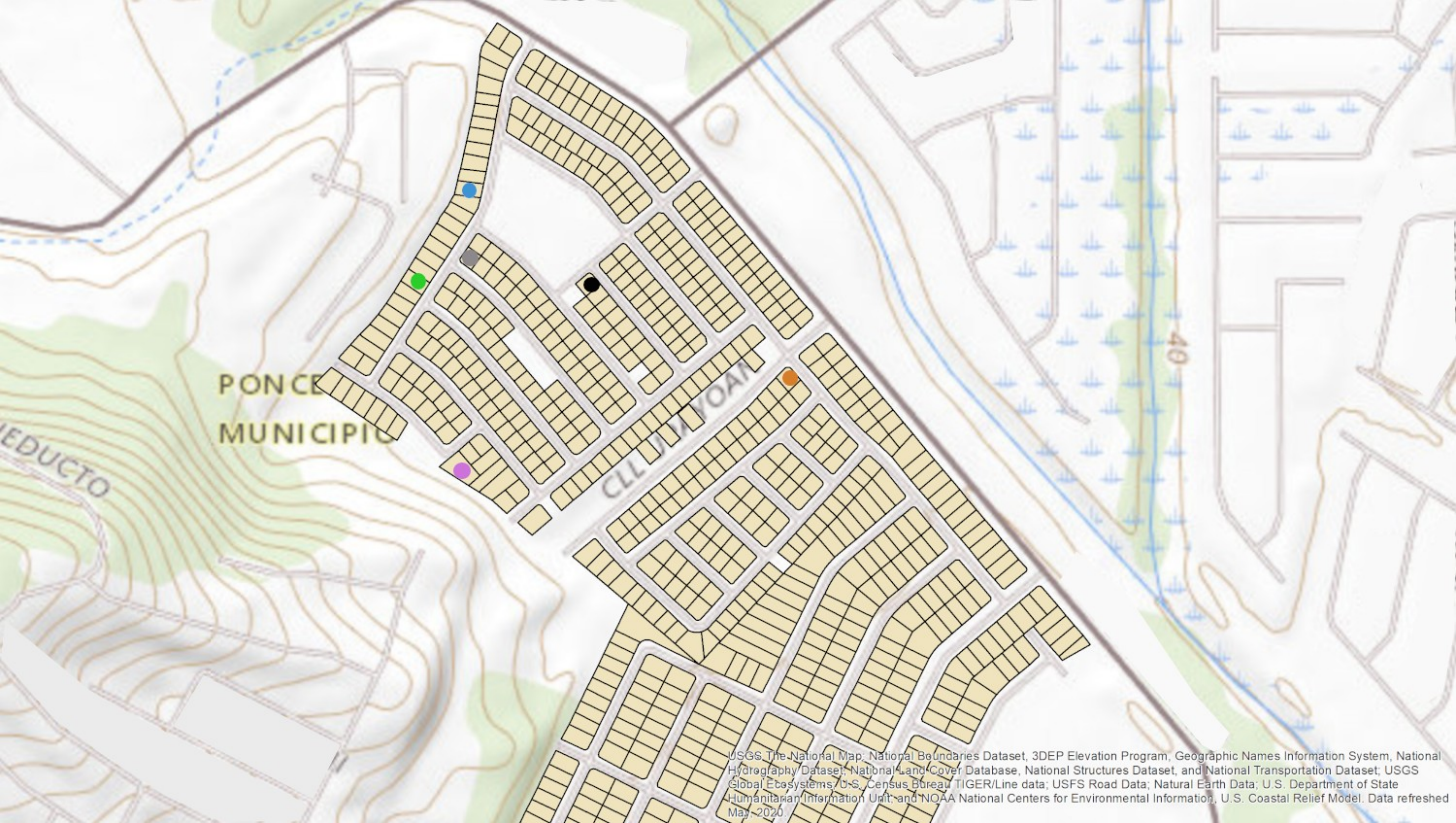
Representation of HTrack’s main map and navigation area. This map recreates how HTrack’s main map is seen upon usage. Street names have been removed to protect participant privacy.

HTrack’s user interface is composed of four tabs: First, a “Main” tab that shows an interactive map paired to a data table, and several fields for data entry (Fig 1). This tab is the primary area for data capture, navigation and location visualization in HTrack. For purposes of this manuscript, the main map area was masked from Fig 1, and a visualization of the map is shown in Fig 2 (map base layers were created and reprinted with permission from USGS The National Map using ArcGIS® software by Esri. ArcGIS® and ArcMap™ are the intellectual property of Esri, are used herein under license and are copyright © Esri, all rights reserved).

**Fig 3:**
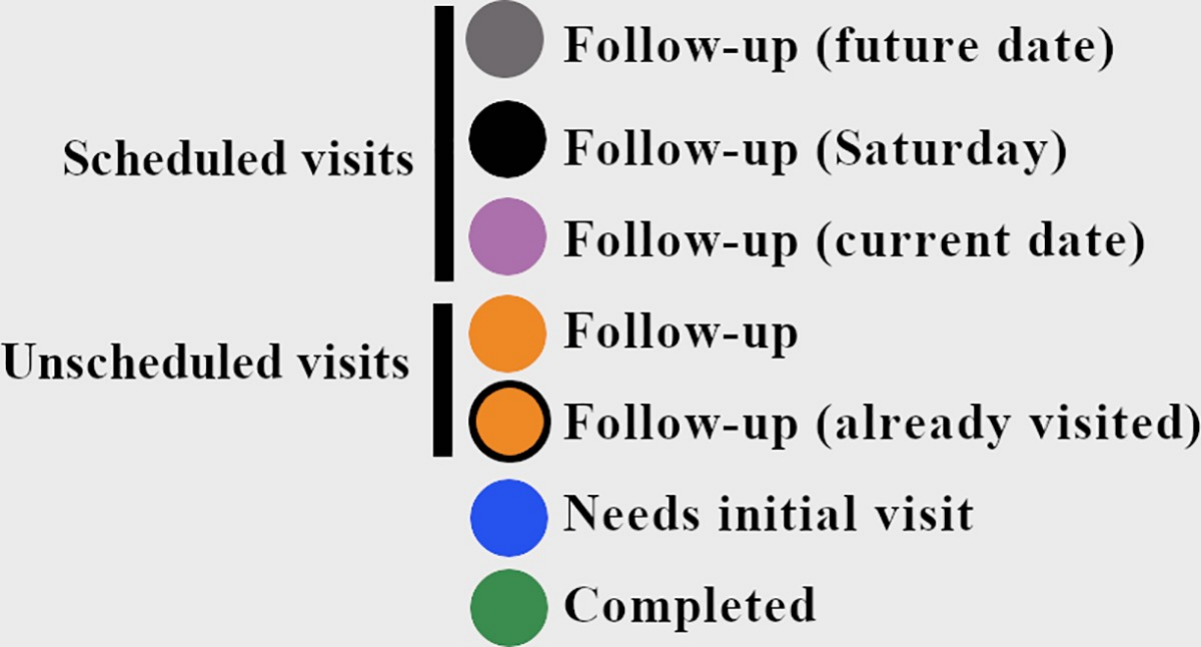
HTrack color-scheme. Blue markers were used for structures that needed an initial visit. Orange markers were used for structures that required unscheduled follow-up visits (a black outline indicated that the structure had already been visited for the day, but still required a follow-up visit). Among scheduled visits, black markers were used for visits scheduled on a Saturday. Grey markers were used for visits scheduled on a weekday. Pink was used for homes that had a visit scheduled for that specific day. Green was used for homes that required no more visits, regardless of recruitment status.

Field staff organized their daily activities by following a visit status color-scheme (Fig 3). The color-scheme is based on the visit details captured; whenever a new visit attempt is saved, the house marker on the map changes color and the data table automatically updates. The paired data table (R package DT [25]) contains household coordinates associated with each HHID and other columns that are populated when the user enters data through the data entry area.

**Fig 4:**
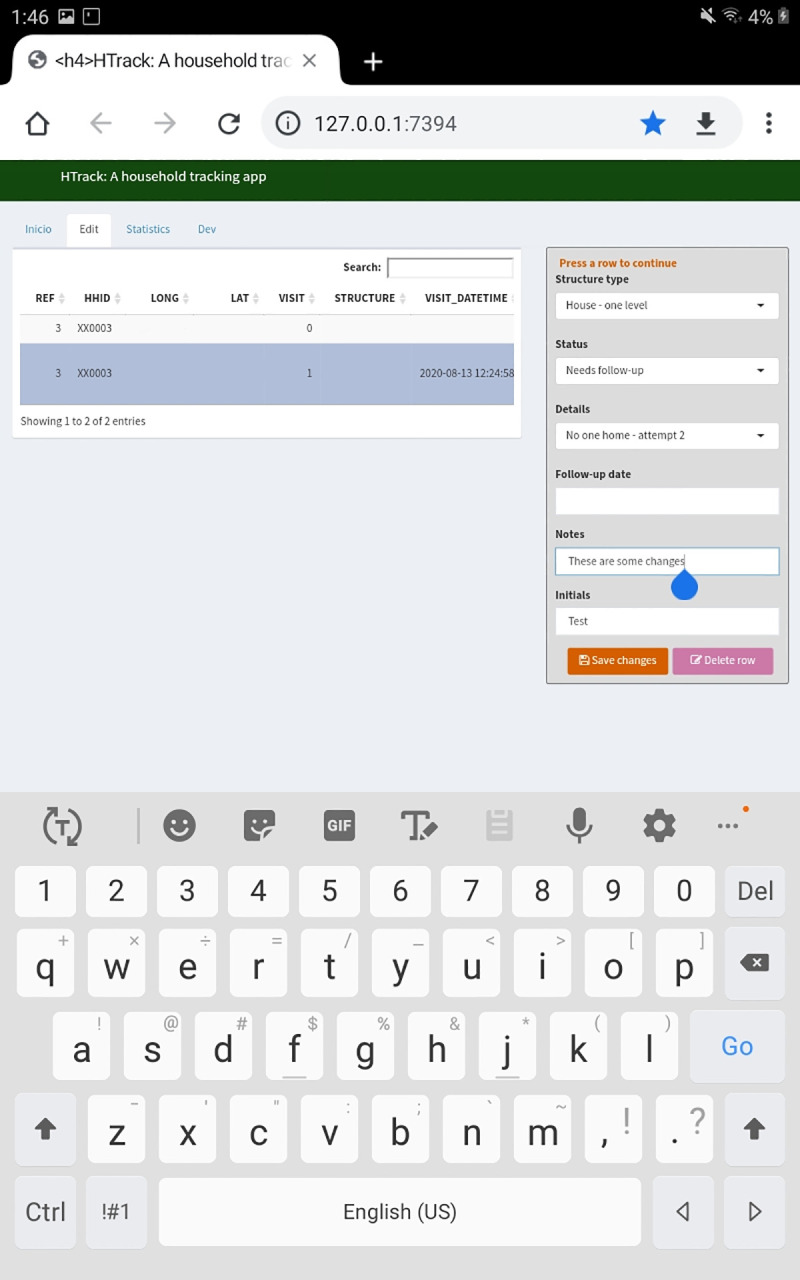
HTrack edit tab serves as the area to perform any changes to the data already entered. This tab allows the user to edit several rows of data after saving. The initial visit row cannot be edited or deleted (visit 0). Coordinates are removed from the image to protect participant privacy.

**Fig 5:**
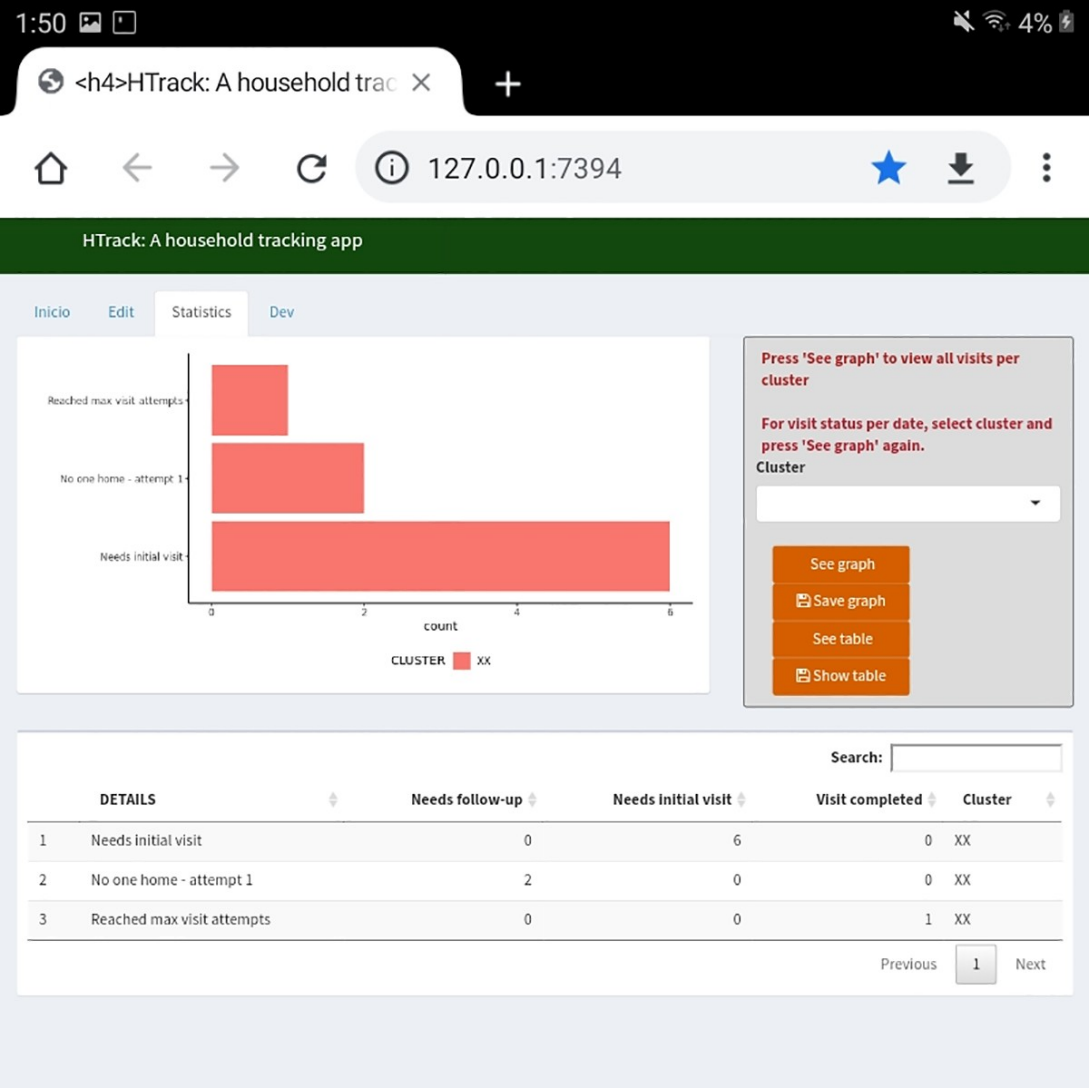
HTrack statistics tab serves as the data visualization area to monitor daily field progress. This area shows the current progress by cluster or overall. Graphs and tables can be downloaded as needed.

The second tab is called “Edit” (Fig 4) and also shows a data table and a data entry area where users can edit information previously entered in the “Main” tab. The third tab, “Statistics”, shows general counts per cluster and graphs to monitor house tracking (Fig 5). These graphs and tables can be downloaded at any time. Finally, the “Developers” tab incorporates new geographic selections, if needed.

### Ethics statement

The Institutional Review Boards at the Centers for Disease Control and Prevention and Ponce Research Institute/Ponce Health Sciences University (PHSU) approved the study protocol. All adult participants provided written informed consent; for minors, parents or guardians provided written consent. For minors 7 to 20 years old, verbal assent was also obtained.

### Data collection, data saving and COPA logistics

Households eligible for enrollment had at least one inhabitant ≤50 years of age. In neighborhood areas, each selected structure was visited a total of three times with at least one visit performed on a Saturday, or until a successful contact leading to a final outcome (recruited, refused, ineligible, not a home, no contact) was made. The user was able to navigate the field through the leaflet map, which used the encrypted tablet’s built-in GPS component once the user selected the community where they were visiting households and recruiting participants. In contrast, recruitment strategies in public housing complexes followed a convenience sampling approach. Here, the staff would coordinate with public housing administrators and select a specific date and centralized location for recruitment attempts. House members would be recruited in order of arrival until target participant numbers were reached.

To collect and save data for each specific visit, the user had to select a house marker in the map and enter the data in all required fields. The required fields used for the COPA project are specified in Table 1. Here, an auto-generated reference column would link the leaflet marker to the main data table. Preloaded data included the HHID and their specific coordinates. The user selected the structure type, status of the visit attempt, details related to the visit status selected, any specific notes and follow-up dates, if needed, and the user initials. Finally, the visit attempt number, and date were automatically updated and saved for each visit. Information captured from participating homes was validated with consent documents and participant-level questionnaires. Data was saved locally in the main HTrack folder as well as in an archive folder with a date stamp. Weekly data files were transferred from the encrypted tablets to secured network folders.

**Table 1:** Data table fields used for the COPA project.

Parameter	Description	Data type or value
**REF**	(auto generated) Back-end reference number. Feeds and links leaflet marker to data table.	Numeric
**HHID**	(preload) Structure ID	Character
**CLUSTER**	(preload) Community initials	Character
**LONG**	(preload) Longitude	Numeric
**LAT**	(preload) Latitude	Numeric
**VISIT**	(auto generated) Visit number. Auto populates as data is saved	Numeric
**STRUCTURE**	(required) The type of structure visited.	character; drop down field
**STATUS**	(required) The final status of the visit being recorded; customizable by project	character; drop down field
**DETAILS**	(required, conditioned) The details of the visit status; values depend on the VISIT_STATUS selected; customizable by project	character; conditioned drop down field
**VISIT_DATETIME**	(auto generated) The system date when the data is saved	Datetime
**FU_DATE**	(optional) A date for a follow up visit, if needed or requested. Chosen through a calendar pop-up	Date
**FU_NOTES**	(optional) Any field-related notes from the user	Character
**INITIALS**	(required) User initials	Character

A description of the data variables used for the data table in the main tab of the app. The main preload includes the HHID and coordinates for each home, while the rest of the variables are populated upon data saving. Several variables are required, and data saving is conditioned on these.

### Interaction with other applications

For COPA, the participant questionnaire was developed in Epi Info. Once a house was selected in the data table, the user had the option to press a button in HTrack that automatically opened that structure’s record in Epi Info, reducing time and data entry errors when searching for the correct record in the Epi Info app to start survey data collection.

## Results

### COPA logistics and change requests

**Fig 6:**
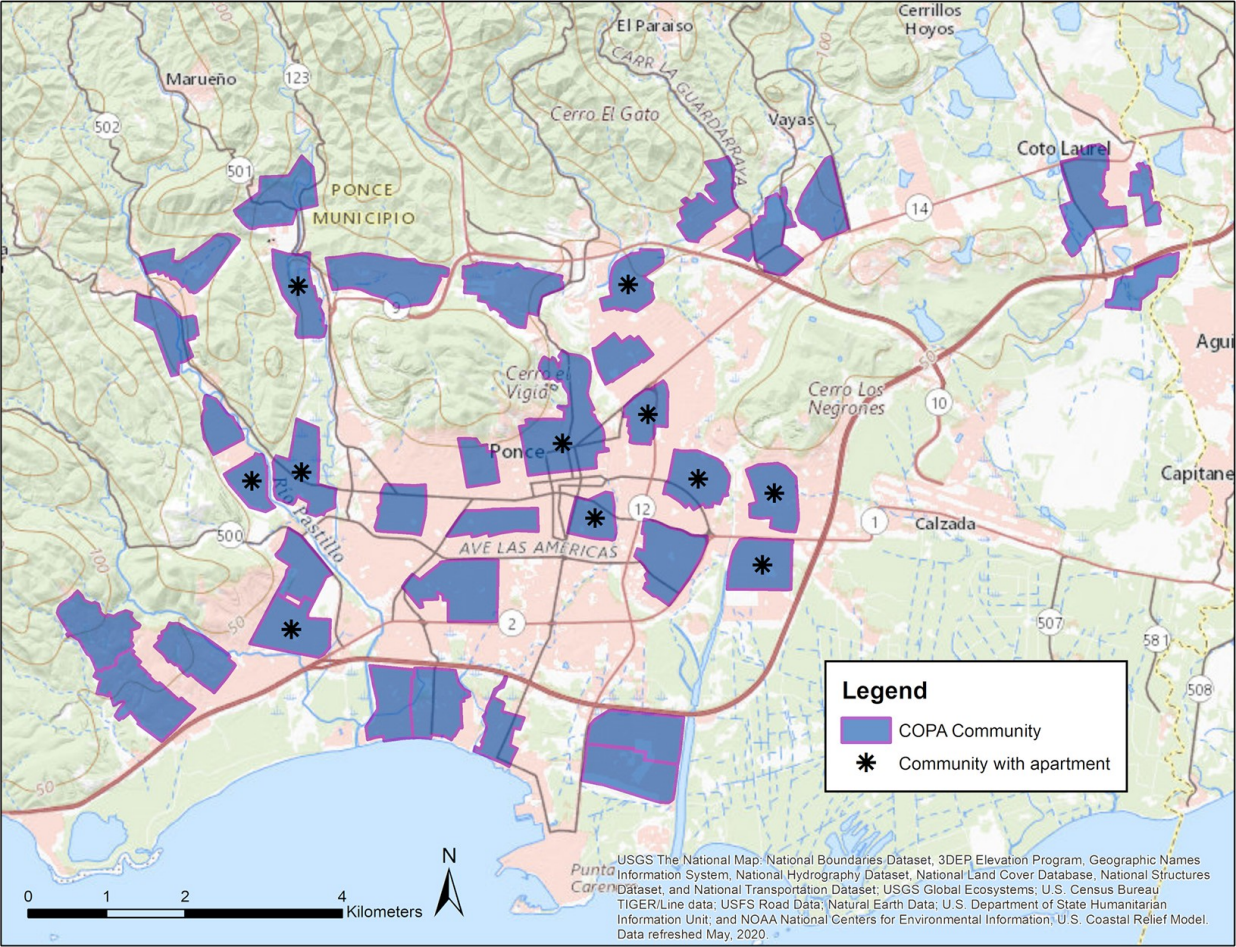
COPA communities. COPA communities, including all 38 clusters, are shown in light blue. Communities with asterisks indicate communities with apartment buildings.

During April 2018–May 2019, HTrack was used by 30 field staff to visit randomly selected homes in 38 communities and capture information about the structure type, eligibility criteria, and field notes. Fig 6 shows a map with each of the communities, where communities containing public housing complexes are marked with an asterisk.

During the first three weeks of the project, the staff provided feedback to improve data collection in the field, which were developed and applied within a week. Changes requested were focused on the visit detail options, which would directly trigger the house marker color-scheme in the main map area. Initially, the main visit details driving the color-scheme involved only three colors: 1) blue for homes that had never been visited by the staff, 2) orange for homes requiring a follow-up visit of any type, and 3) green for homes not needing additional visits. However, this color-scheme proved difficult for the staff given the different types of follow-up visits required, which included scheduled and unscheduled follow-up visits, as well as Saturday visits. In response, new colors and outlines were added to assist in tracking of scheduled and unscheduled visits and the final color-scheme used for COPA is shown in Fig 3. Structures that had not been visited remained blue. Unscheduled visits remained orange and would be outlined if they were already visited for the day. When house members requested a visit at a later date or the staff wanted to visit the home at a later date, they could schedule a follow-up visit using the calendar option in the HTrack data entry area. Homes scheduled during weekdays were marked as gray. If the scheduled visit was on the current day (the tablet’s system date), they showed as pink, enabling the staff to prioritize these homes over any other structure. Any homes with an overdue visit date were outlined in red. Regardless of visit type (scheduled or unscheduled), if no contact was made on two occasions and the house had not been visited on a Saturday, a message stating that the next visit should be on a Saturday appeared and the house marker turned black. The staff could ignore any black house markers during weekday visit attempts. Finally, recruited homes or homes that had no more pending visits were shown in green.

The use of HTrack was limited in apartment building complexes. In these areas, the markers are tightly grouped and there is a limited zoom capability from the offline map tiles generated for the app, affecting the selection of the correct house markers. To overcome this limitation, we developed an additional feature that allowed the staff to select the HHID from the data table, which isolated that house marker in the map and allowed the staff to begin data entry.

### COPA visit outcomes

A total of 45,904 visits were made with HTrack, including 6,378 (14%) scheduled and 39,526 (86%) unscheduled visits. For simplicity, we grouped the 38 communities into two main categories to provide aggregate level statistics for apartment complexes and neighborhood areas (Table 2). Among 23,830 randomly selected homes and apartment building units, a total of 4,090 participants and 9.7% homes were recruited. Among other homes visited, 37.4% were not eligible, 25.6% were visited three times but no contact was made, 18.8% were classified as not being homes or vacant homes, and 8.5% of homes refused participation. Ineligible participants were those that did not fall within the age category required for the COPA study, those who slept less than 4 nights at the home, or those that were moving in the next 6 months. The average number of houses recruited per community was 61 after a minimum of 6.4 weeks in each community.

**Table 2:** Final visit outcomes among households visited.

Type of area	Visited	Recruited	Refusals	Not eligible	No contact	Not a home
	N	n (%)	n (%)	n (%)	n (%)	n (%)
Neighborhood areas	23182	1829 (7.9)	2012 (8.7)	8796 (37.9)	6071 (26.2)	4474 (19.3)
Apartment complexes	648	489 (75.5)	4 (0.6)	117 (18.1)	38 (5.9)	0 (0)
**Total**	23830	2318 (9.7)	2016 (8.5)	8913 (37.4)	6109 (25.6)	4474 (18.8)

Final visit outcomes obtained for the COPA project in 2018.

## Discussion

This paper presents a R-Shiny application, HTrack, which was developed for field navigation, visit tracking, and data capture of field activities, following data security requirements as established for the COPA project. Though other software incorporate these features, none include the full capabilities that HTrack offers, including built-in offline navigation features, field visit tracking, and direct linkage and integration with external software, while still maintaining data security requirements. For COPA, we show that HTrack facilitated rapid and accurate electronic data capture in a secure offline setting. This application provided near real-time data crucial for assessing field logistics and workflows. All outcomes obtained from HTrack allowed for weekly project monitoring. Compared with paper-based data collection, HTrack saved staff time and resources by reducing printing costs, reducing data entry delays, eliminating the need for detailed printed maps across a large geographic area, and simplifying the systems needed for project management.

The source code for this app is written so that it can be customized as needed for different field studies and strategies. This enables versatility and speed for any changes or additions to the visit outcome selections, color-scheme, and descriptive data portrayed, during app testing or implementation. HTrack can be further developed to suit specific study needs. For example, newly developed HTrack instances used solely for apartment building complexes may provide for a more robust approach since the R leaflet package allows for custom-made maps–in this case, apartment building schematics. HTrack can also be customized for other purposes, such as navigation-only or to capture GPS coordinates from any location of interest, even without a preload file.

In general, any study requiring field-level sampling, navigation and visit tracking can greatly benefit from HTrack, particularly in settings without internet access and where strict data security protocols are needed.
